# High resolution ultrasound-guided microinjection for interventional studies of early embryonic and placental development *in vivo *in mice

**DOI:** 10.1186/1471-213X-6-10

**Published:** 2006-02-27

**Authors:** John C Slevin, Lois Byers, Marina Gertsenstein, Dawei Qu, Junwu Mu, Nana Sunn, John CP Kingdom, Janet Rossant, S Lee Adamson

**Affiliations:** 1 Samuel Lunenfeld Research Institute of Mount Sinai Hospital, Toronto, Canada; 2 Department of Obstetrics and Gynecology, University of Toronto, Toronto, Canada; 3 Department of Physiology, University of Toronto, Toronto, Canada; 4 Department of Medical Genetics and Microbiology, University of Toronto, Toronto, Canada

## Abstract

**Background:**

In utero microinjection has proven valuable for exploring the developmental consequences of altering gene expression, and for studying cell lineage or migration during the latter half of embryonic mouse development (from embryonic day 9.5 of gestation (E9.5)). In the current study, we use ultrasound guidance to accurately target microinjections in the conceptus at E6.5–E7.5, which is prior to cardiovascular or placental dependence. This method may be useful for determining the developmental effects of targeted genetic or cellular interventions at critical stages of placentation, gastrulation, axis formation, and neural tube closure.

**Results:**

In 40 MHz ultrasound images at E6.5, the ectoplacental cone region and proamniotic cavity could be visualized. The ectoplacental cone region was successfully targeted with 13.8 nL of a fluorescent bead suspension with few or no beads off-target in 51% of concepti microinjected at E6.5 (28/55 injected). Seventy eight percent of the embryos survived 2 to 12 days post injection (93/119), 73% (41/56) survived to term of which 68% (38/56) survived and appeared normal one week after birth. At E7.5, the amniotic and exocoelomic cavities, and ectoplacental cone region were discernable. Our success at targeting with few or no beads off-target was 90% (36/40) for the ectoplacental cone region and 81% (35/43) for the exocoelomic cavity but tended to be less, 68% (34/50), for the smaller amniotic cavity. At E11.5, beads microinjected at E7.5 into the ectoplacental cone region were found in the placental spongiotrophoblast layer, those injected into the exocoelomic cavity were found on the surface or within the placental labyrinth, and those injected into the amniotic cavity were found on the surface or within the embryo. Following microinjection at E7.5, survival one week after birth was 60% (26/43) when the amniotic cavity was the target and 66% (19/29) when the target was the ectoplacental cone region. The survival rate was similar in sham experiments, 54% (33/61), for which procedures were identical but no microinjection was performed, suggesting that surgery and manipulation of the uterus were the main causes of embryonic death.

**Conclusion:**

Ultrasound-guided microinjection into the ectoplacental cone region at E6.5 or E7.5 and the amniotic cavity at E7.5 was achieved with a 7 day postnatal survival of ≥60%. Target accuracy of these sites and of the exocoelomic cavity at E7.5 was ≥51%. We suggest that this approach may be useful for exploring gene function during early placental and embryonic development.

## Background

In utero microinjection of mouse embryos has proven valuable for exploring the developmental consequences of altering gene expression using adenoviral or retroviral vectors [1-12], or for injecting cells to study cell lineage or migration [13,14]. Microinjection is also a useful approach for rescuing mutant embryos to validate gene therapies and/or circumvent embryonic morbidity or mortality thereby permitting study of a gene's role later in development or adulthood [15]. This approach has been largely limited to studying embryos at embryonic day 9.5 (E9.5) or greater when the uterus and decidua have become thinner and the placenta and embryo are relatively large so that ultrasound or trans-illumination can be used to guide injections into the conceptus in the exteriorized uterus. However, knocking out or mutating genes critical for early development of the yolk sac, chorioallantoic placenta, hematopoietic system, embryonic heart and vasculature often cause embryonic lethality before E9.5 [16-18]. Thus, the goal of the current study was to develop and validate methods to accurately target specific regions of the conceptus under high-resolution ultrasound guidance at E6.5 to E7.5 of gestation. At this stage, the embryo is not yet dependent on placental or circulatory function for survival. We show that ultrasound-guided microinjection into the ectoplacental cone region at E6.5 or E7.5 and the amniotic cavity at E7.5 was achieved with a 7 day postnatal survival of ≥60% and that target accuracy of these sites and of the exocoelomic cavity at E7.5 was ≥51%, suggesting this method will provide a feasible approach for future studies.

## Results and discussion

### Ultrasound imaging of the early embryo

**Figure 1:**
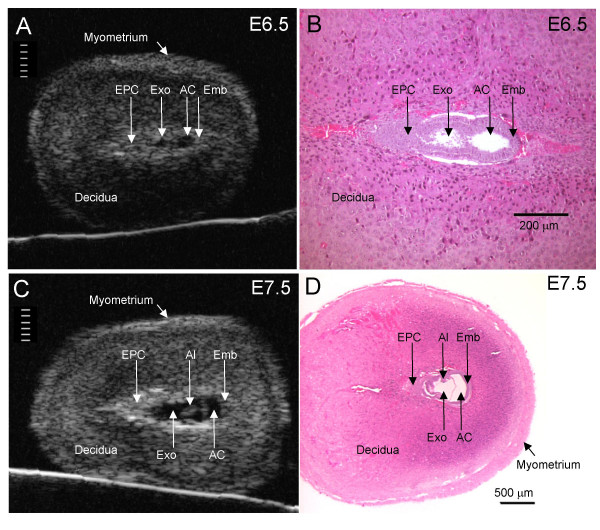
**Anatomical detail visible in ultrasound images**. Ultrasound images (A, C) and H&E histological sections (B, D) of implantation sites at E6.5 (A, B) and E7.5 (C, D). Divisions in the scale in A and C are 100 μm apart. The conceptus in histological sections is smaller than in vivo due to shrinkage during tissue preparation (fixation and dehydration). AC, amniotic cavity; Al, allantois; Emb, embryo; EPC, ectoplacental cone region; Exo, exocoelomic cavity.

We used a 40 MHz ultrasound transducer to image the conceptus within the exteriorized uterus in isoflurane-anesthetized pregnant mice, and compared results with histological images of the conceptus obtained at the same gestational ages (figure 1). At E6.5, a relatively echogenic (bright) ectoplacental cone region and a small echolucent (dark) cavity containing the embryo was visualized inside a ~1.5 mm thick decidua. The 3 dimensional (D) impression of the E6.5 conceptus in real-time (additional file 1) was clearer than was evident from 2D still images (figure 1A). The most prominent structure in the conceptus at this stage was the cone-shaped ectoplacental cone region where embryonic trophoblast cells are invading the mesometrial maternal decidua and where the future placenta will develop. This region is bordered by echogenic calcium deposits [19] making it an easily identified target on ultrasound. The diameter of the proamniotic cavity containing the embryo was ~150 μm at E6.5, which was near the limit of resolution of the ultrasound transducer (50 μm [20]) and the outer diameter of the microinjection pipette (50–80 μm). Thus, only the larger echogenic ectoplacental cone region presented a feasible target at this gestational age.

At E7.5, the amniotic and exocoelomic cavities, and the ectoplacental cone region were each discernable on ultrasound (figure 1C). When compared to an appropriate histological section at the same gestation (figure 1D), the structural detail in the ultrasound image can be appreciated. As at E6.5, the 3D impression of the E7.5 conceptus observed in real-time (additional file 2) was clearer than in 2D still images (figure 1C).

### Dissection to establish conditions for accurate bead microinjection

The maximum injection volume that resulted in few or no beads off-target was determined in 4 mice at E7.5 as follows. We began by microinjecting an aqueous suspension of 3 μm diameter green fluorescent beads into the amniotic cavity at a volume of 207 nL, which was approximately 50% of the lowest previously published volume [1]. This caused marked distension of the cavity and surrounding area so we gradually reduced the volume to 13.8 nL in successive experiments. Injection accuracy and frequency of beads in adjacent off-target areas was visually evaluated using a stereomicroscope in implantation sites that were dissected as previously described [21] within a few hours following microinjection.

**Figure 2:**
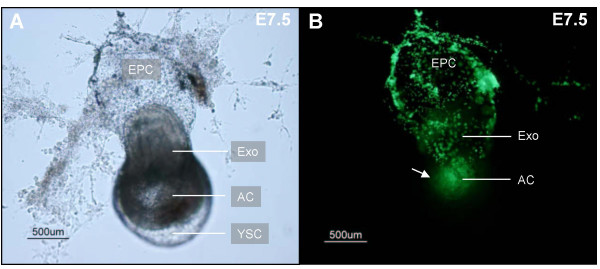
**Off-target beads following 69 nL microinjection into the amniotic cavity**. This E7.5 conceptus was dissected a few hours after ultrasound-guided microinjection of a 69 nL volume containing 3 μm diameter fluorescent beads into the amniotic cavity. (A) The amniotic and yolk sac cavities were visibly distended (e.g. compare to figure 3D) when viewed under a dissection microscope. (B) When the same embryo was viewed under a fluorescent microscope, beads were visible within the amniotic cavity (arrow) but were also present in the adjacent exocoelomic cavity as well as in the ectoplacental cone region. AC, amniotic cavity; EPC, ectoplacental cone region; Exo, exocoelomic cavity; YSC, yolk sac cavity.

We found that microinjection volumes of 69 nL or more into the E7.5 amniotic cavity visibly distended the cavity. At necropsy, the injected 3 μm diameter fluorescent beads were frequently found in adjacent off-target cavities (figure 2A, B). This is consistent with our volume estimate of ~50 nL for the embryo and both cavities (based on an ellipsoid of approximately 0.3 mm diameter × 1 mm length). Distension and beads in adjacent off-target regions were minimal when a volume of 13.8 nL was injected at a rate of 23 nL/s.

**Figure 3:**
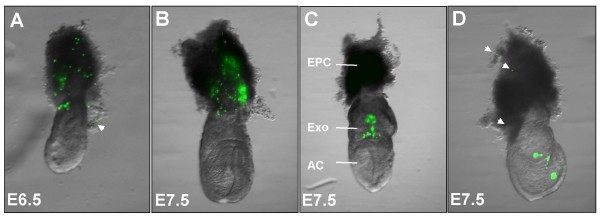
**Target accuracy**. Concepti dissected a few hours after ultrasound-guided microinjections of a 13.8 nL volume containing 3 μm diameter fluorescent beads. In these examples, there were few (e.g. arrows in A&D) or no beads considered off-target (e.g. B&C). The ectoplacental cone region was targeted in (A) at E6.5 and (B) at E7.5. At E7.5, the exocoelomic cavity (C) and amniotic cavity (D) were also targeted. AC, amniotic cavity; EPC, ectoplacental cone region; Exo, exocoelomic cavity.

With a suitable injection volume of 13.8 nL established, we next evaluated the accuracy of targeting the amniotic cavity (additional file 3), exocoelomic cavity (additional file 4), and the ectoplacental cone region (additional file 5) at E7.5 (table 1). The accuracy of targeting the ectoplacental cone region at E6.5 was similarly determined (table 1). A few hours following microinjection, mice were euthanized then dissected under a stereomicroscope and the concepti placed in a 96-well plate filled with calcium and magnesium-free phosphate-buffered saline (PBS). Each conceptus was examined under a stereomicroscope and the location of fluorescent beads recorded (figure 3). Less than 10% of injected concepti were damaged during dissection and were excluded from analysis. Greater than 90% of concepti contained beads (table 1) but they were not always confined to the target. Microinjections where many beads were off-target were excluded from the "on target" count because they were considered inaccurate even if some beads were in the targeted location. Examples of "on target" microinjections are shown in figure 3.

**Table 1:** Target accuracy following micro-injection.

**Age**	**Target**	**Necropsy**	**Mice**	**Concepti**^1^	**Beads in conceptus^2^**		**Beads localized in target^3^**		
			#	#	#	%	#	%	95% CI

E6.5	EPC	Within 24 hours	5	55	50	91	28	51*	38–64
E7.5	EPC	Day of injection	4	40	38	95	36	90	77–96
E7.5	AC	Day of injection	4	50	47	94	34	68	54–79
E7.5	Exo	Day of injection	4	43	40	95	35	81	68–91

1 number of concepti analyzed (<10% were damaged during dissection and were not included)

2 number of concepti containing beads at one or more sites

3 number of concepti with beads localized in target with few or no beads off-target

* significantly different than EPC at E7.5

EPC = ectoplacental cone region, AC = amniotic cavity, Exo = exocoelomic cavity, CI = confidence interval

**Figure 4:**
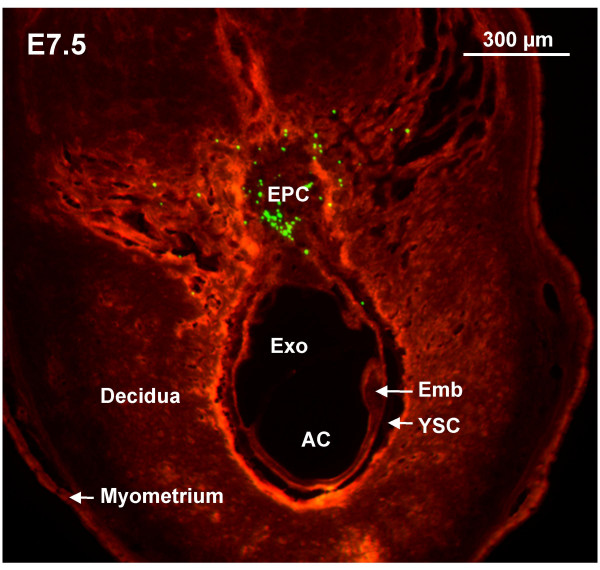
**Histological detection of injected beads**. Implantation site (tissue autofluoresces orange) containing green fluorescent beads collected a few hours following ultrasound-guided microinjection into the ectoplacental cone region at E7.5. Beads visualized in 50 μm frozen sections were primarily localized to the targeted ectoplacental cone region. AC, amniotic cavity; Emb, embryo; EPC, ectoplacental cone; Exo, exocoelomic cavity; YSC, yolk sac cavity.

At E6.5, a volume of 13.8 nL microinjected at a rate of 23 nL/s into the ectoplacental cone region resulted in beads being present in 91% of concepti (50/55) (table 1). However, beads were specifically localized to the ectoplacental cone region in only 51% of those injected (28/55). Use of a smaller injection volume may improve accuracy at this age. Targeting accuracy of the ectoplacental cone region was significantly improved at E7.5 (i.e. no overlap in 95% confidence intervals relative to survival at E6.5). At E7.5, beads were localized to the ectoplacental target in 90% of concepti (36/40) (table 1) (figure 3A, B). In 50 μm frozen tissue sections at E7.5, fluorescent beads were visible in the ectoplacental cone region (figure 4) suggesting that beads could be used in future studies to mark the site of microinjection in histological sections [22]. At E7.5, we were also able to target the exocoelomic and amniotic cavities. Our success at targeting with few or no beads off-target was 81% for the exocoelomic cavity and 68% for the smaller amniotic cavity (table 1, figure 3C, D).

### Assessment of embryonic survival and effect of development on localization of beads

Embryonic survival following microinjection was determined up to 1 week following birth (table 2). Between E7.5–18.5, mice injected at E6.5 were euthanized, the uterus removed, and the individual embryos dissected. Embryos with normal coloration, and without evidence of autolysis were considered to have survived. Additional mice were allowed to progress to delivery. The number of live pups on the day of birth (either at cesarean section or following spontaneous delivery), and at one-week post delivery was determined. The number of survivors relative to the number of injected sites was determined for each mouse. The localization of beads was determined up to 7 days post microinjection. Determination at later time points was more difficult because of rarefaction of the beads due to growth and development of the tissue mass containing the beads, and the inability to detect beads embedded within the embryo or placenta due to increased tissue opacity.

**Table 2:** Survival following microinjection or sham procedures.

age at intervention	E6.5	E7.5	E7.5	E7.5	E7.5	E7.5
target	EPC	EPC	Exo	AC	Sham 1	Sham 2
**Embryo 2–12 d postinjection**						
# mice	10	8	6	15	6	nd
# concepti	119	76	64	145	90	nd
# alive	93	68	50	98	60	nd
% alive	78	89 †	78	68	67	nd
95% CI for % alive	70–85	80–95	66–87	59–75	56–76	nd
**At birth**						
# mice	4	nd	nd	3	5	5
# concepti	56	nd	nd	43	61	62
# alive	41	nd	nd	26	34	52
% alive	73	nd	nd	60	56 *	84
95% CI for % alive	60–84	nd	nd	40–75	42–68	72–92
**1 wk Postnatal**						
# mice	4	2	nd	3	5	5
# concepti	56	29	nd	43	61	62
# alive	38	19	nd	26	33	52
% alive	68	66	nd	60	54 *	84
95% CI for % alive	54–80	46–82	nd	44–75	41–67	72–92

nd = not done; # = number; CI = confidence interval; EPC = ectoplacental cone region; Exo = exocoelemic cavity; AC = amniotic cavity; Sham 1 = surgical procedure and ultrasound imaging without microinjection; Sham 2 = brief surgery to count embryos; † = % alive significantly greater than for AC and Sham 1; * = % alive in Sham 1 is significantly less than in Sham 2

#### Ectoplacental cone region

Following microinjection into the ectoplacental cone region at E6.5, 78% of embryos in 10 mice were still alive when assessed 2 to 12 days later, 73% of pups from 4 mice were still alive at birth, and 68% were still alive at 1 week of age (table 2). In 7 of the 10 mice evaluated during pregnancy, the conceptus was dissected within 7 days post injection and the localization of beads was assessed by gross morphology. 74% (62/84) of the embryos were alive of which 95% (59/62) had beads in the conceptus. 95% (56/59) of these had beads predominantly localized to the placental region.

**Figure 5:**
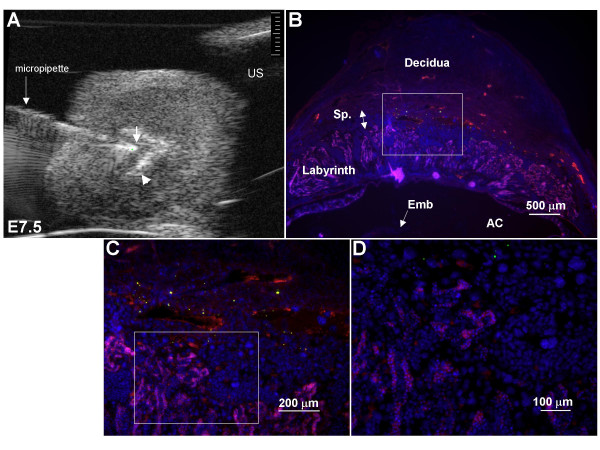
**Localization of fluorescent beads in the spongiotrophoblast layer following ectoplacental cone microinjection**. (A) Placement of the micropipette tip (arrow) near the center of the ectoplacental cone region (EPC) at E7.5. The EPC is demarcated by an echogenic 'V'-shape (arrowhead) [19], and is therefore easy to identify on ultrasound. (B-D) Histological images obtained from a conceptus at E11.5 following microinjection of green fluorescent beads into the EPC at E7.5. Frozen sections were counterstained with DAPI (nuclei stain blue) and immunofluorescence was used to detect collagen 4 in the basement membrane of the labyrinth capillaries (pink). The beads are localized in the placental spongiotrophoblast layer between the labyrinth and the decidua. Boxed regions in (B) and (C) are shown as higher power images in (C) and (D) respectively. AC, amniotic cavity; Emb, embryo; Sp, spongiotrophoblast layer; US, uterine stabilizer.

Following microinjection into the ectoplacental cone region at E7.5, 89% of embryos in 8 mice were still alive when assessed 2–12 days later (table 2). In 6 of these mice, the localization of beads was determined by gross morphology. 79% (55/70) of the embryos survived of which 96% (53/55) had beads in the conceptus. 94% of these (50/53) had beads predominantly localized to the placental region. Histology was performed on 7 concepti with surviving embryos from one pregnant mouse at E11.5 to determine the location of beads more precisely using 50 μm frozen sections. The vast majority of injected beads were localized in the placental spongiotrophoblast layer (figure 5B–D). A few beads were often observed between the yolk sac membranes, and a few beads were occasionally observed in the labyrinth or amniotic cavity. Localization of beads within the spongiotrophoblast layer is consistent with the ectoplacental origin of cells in this layer [24]. 66% of pups of 2 mice were still alive at 1 week after birth. Surviving neonates had no apparent abnormalities in morphology or body size.

#### Exocoelomic cavity

**Figure 6:**
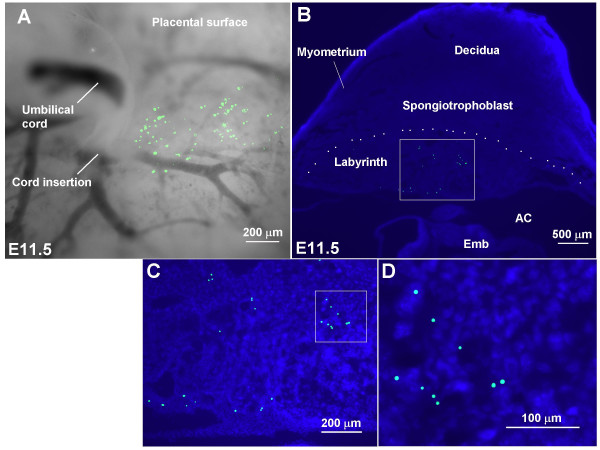
**Localization of fluorescent beads in the placental labyrinth following exocoelomic microinjection**. Images of placentas dissected at E11.5 following ultrasound-guided microinjection of green fluorescent beads into the exocoelomic cavity at E7.5. (A) Stereomicroscopic image showing fluorescent beads embedded in the fetal surface of the placenta near the cord insertion. (B) 50 μm frozen section through the placenta and implantation site showing fluorescent beads distributed within the labyrinth layer, extending as deep as the border between the labyrinth and spongiotrophoblast layers (dotted line). Boxed regions in (B) and (C) are shown as higher power images in (C) and (D) respectively. Cell nuclei were stained with DAPI in B, C, and D. AC, amniotic cavity; Emb, embryo.

When we performed microinjection into the exocoelomic cavity at E7.5, 78% of embryos in 6 mice were still alive when assessed 2–12 days later (table 2). The localization of beads in 4 of these 6 mice was assessed in concepti with surviving embryos by gross morphology. 90% (45/50) had beads in the conceptus and in all cases the majority of beads were present in the placental region. While assessing the location of the beads we noted that the fluorescent beads were visible, trapped on the placental surface (figure 6A). In 2 of these mice we further assessed the location of the beads within the placenta by performing 50 μm frozen sections at E11.5 and found the fluorescent beads to be distributed deep within the placental labyrinth in cross-sections (figure 6B–D). This was observed in 80% (21/26) of placentas from surviving embryos in the 2 mice that were assessed. Possibly beads within the exocoelomic cavity and/or beads adherent to the allantois or chorionic surfaces following injection into this cavity became trapped following allantoic attachment and subsequent invasion into the chorion, and by this means were carried deep into the developing labyrinth during chorio-allantoic morphogenesis [23,24].

#### Amniotic cavity

**Figure 7:**
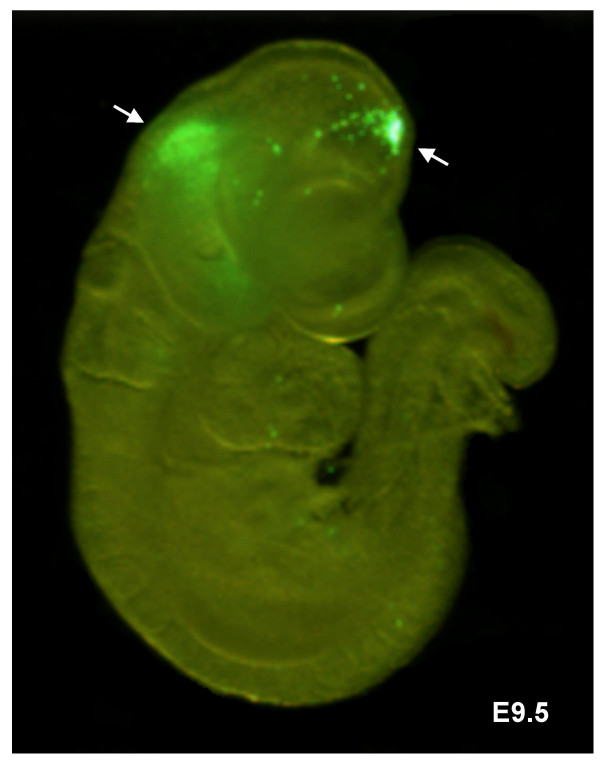
**Embryonic localization of fluorescent beads following amniotic cavity microinjection**. E9.5 embryo dissected 2 days after ultrasound-guided microinjection of 13.8 nL of fluorescent beads into the amniotic cavity. Green fluorescent beads were visible within the neural system (arrows) as well as on the skin surface. The embryo was imaged using a Leica MZ 16FA stereomicroscope with GFP filter.

We assessed survival after microinjection of the amniotic cavity at E7.5 in 15 mice. 68% of embryos were still alive when assessed 2–12 days post injection. The distribution of beads was assessed in 33 embryos from 3 pregnant mice 2 days following microinjection into the amniotic cavity at E7.5. 82% (27/33) survived and all had beads within the conceptus. 62% (17/27) of the surviving embryos were found to have fluorescent beads on their skin and deep within their structure. The deeper beads may have become trapped within the neural tract during neural tube closure (figure 7). 73% (38/52) of similarly injected surviving embryos examined 4–8 days following microinjection at E7.5 had beads visible within their skin surface. At this stage, tissue opacity made detection of deeper beads difficult to assess. 60% of pups of 3 mice were still alive at birth and at 1 week of age (table 2). Surviving neonates had no apparent abnormalities in morphology or body size.

#### Sham experiments

We performed sham experiments to assess the effect of microinjection on embryo viability. We also determined survival in non-operated control pregnancies. For sham 1, all experimental procedures at E7.5 were replicated except no microinjection was performed (surgical exposure, ultrasound imaging, uterine manipulation, and experimental duration were replicated). For sham 2, uterine horns were exposed at E7.5 to count the number of implantation sites and then were immediately replaced. For normal survival in non-operated control pregnancies, we determined embryonic number as a function of gestational age in control mice without prior interventions. We examined an average of 15 pregnant mice per gestation day from E6.5 to E18.5 at necropsy, and counted the number of viable embryos. Linear regression was used to show that the average number of embryos at E0 was 14 and the rate of embryo loss per day was 0.13. This information was used to calculate the average embryonic survival rate in pregnancies without interventions at specific gestational ages.

In sham 1, 54% of embryos were alive and appeared normal when assessed at 1 week postnatal age (table 2). This rate did not differ significantly from survival rates following microinjection into the amniotic cavity (60%) or the ectoplacental cone region (66–68%) (i.e. there was overlap in 95% confidence intervals) indicating that the embryonic lethality was predominantly due to the surgical procedure not to the microinjection procedure. In sham 2, survival at 1 week postnatal age was 84% which was significantly higher than in Sham 1 (54%) (table 2). Sham 2 survival at birth (84%) was close to the 89% survival expected for non-operated control pregnancies. Thus, optimal embryonic survival depends upon keeping the duration of the surgical procedure short and minimizing uterine manipulation.

## Conclusion

Ultrasound-guided microinjection provides a feasible approach for experimental interventions into either the embryonic (E7.5) or extra-embryonic (E6.5–7.5) regions. Limitations include the presence of beads in off-target locations and a significant increase in embryonic deaths. In the current study we injected fluorescently labeled microspheres in vehicle (0.9% saline). In future studies the vehicle could include inhibitory RNA, transfection vectors, specific antibodies, or labeled cells and thus this method could be used to explore the developmental consequences of altering gene expression, or for studying cell lineage and migration. However, whether the localization of cells, small molecules or solutes would be similar to that of the beads used in the current study is unknown. This limitation may be addressed by microinjecting bioactive agents tagged or absorbed to spheres, or by microinjecting cells that are labeled so that spheres or cells can be localized later either grossly during dissection or in histological sections.

We speculate that microinjections into the exocoelomic cavity may be useful for studies of the development of the yolk sac (e.g. vascularization and hematopoiesis), the interactions between the allantois and chorioallantoic placenta (e.g. chorio-allantoic fusion and morphogenesis) and the development of the placental labyrinth layer. In addition, the ectoplacental cone region can be targeted directly, thereby placing agents among relatively undifferentiated, early placental trophoblasts. Since umbilical blood flow does not commence until approximately E9.5 [20,25], injected agents are likely to remain within this target location and not circulate systemically in the embryo unlike placental injections performed in later gestation. Microinjections into the amniotic cavity may be useful for influencing early events in embryonic development such as gastrulation, axis formation, and neural tube closure.

## Methods

The study protocol was approved by the Mount Sinai Hospital Animal Care Committee, and was conducted in accord with the guidelines of the Canadian Council on Animal Care.

### Animals and animal-related procedures

**Figure 8:**
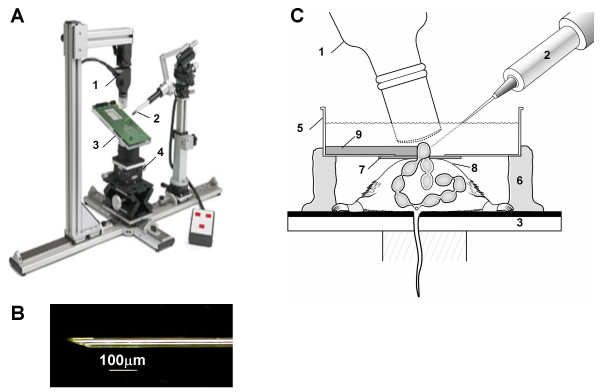
**Microinjector apparatus and experiment set-up**. (A) The rail system maintains the alignment of the image plane of the ultrasound transducer (1) with the trajectory of the microinjector (2). The stage (3) on which the mouse is placed is adjusted to place the target region of the conceptus in the image plane by adjusting the position of the stage using the XYZ controls (4). The rail system increases efficiency by reducing time to target acquisition, improving accuracy and as a result contributes to improved overall survival. (B) The sharp bevelled tip of the microinjection pipette is shown. (C) A modified Petri dish containing PBS (5) is supported above the mouse using Plasticene blocks (6). A segment of the uterus is exposed through a small midline abdominal incision into the Petri dish. A thin transparent rubber membrane (7) attached to the under surface of the Petri dish (5) seals the dish to the dry denuded maternal skin (8), while a thicker silastic membrane (9) submerged within the Petri dish stabilizes the uterus during microinjection.

CD-1 mice (Charles River [28]) were naturally mated. Day 0.5 of gestation was defined as noon on the day a vaginal plug was found after overnight mating. All procedures were carried out in a laminar flow hood. Anesthesia was induced in a chamber then maintained by facemask with 1.5% isoflurane. Body temperature was monitored via rectal thermometer and maintained at 35–38°C using a heating pad and lamp, and heart rate was monitored by electrodes attached to the paws (Indus Instruments [29]). Maternal well-being was carefully monitored to maintain an appropriate body temperature, heart rate, respiratory vigor, and anesthetic depth throughout the procedure. All hair was removed from the abdomen by shaving followed by a chemical hair remover (Nair, Carter-Horner Inc. [30]). The skin was cleansed with 70% ethanol, and the abdomen opened with a 2 cm vertical midline incision along the linea alba (midline avascular region). The stage upon which the mouse was mounted (figure 8) was tilted head down (between 35–45°) to displace the bowel, the uterine horns were gently exteriorized, and the number of implantation sites recorded. The uterine horns were replaced in the abdominal cavity except for a short segment containing 1–3 implantation sites at the ovarian end of the right horn. This segment was positioned just below the skin at the incision site. The maternal skin was re-dried using a small amount of alcohol and a cotton swab.

A modified Petri dish (100 mm diameter, 25 mm deep), similar to that used in earlier work on older embryos [1,2,13,14,26,27], was positioned above the incision site as follows (figure 8C). The Petri dish had a 25 mm hole in the bottom, which was sealed from below by a thin transparent rubber membrane (40 mm square [31]). The membrane had a 10 × 1 mm slit cut in the centre, which was positioned above the skin incision (figure 8C). Fine forceps were passed through the slit in the rubber membrane, the skin edges were gently apposed, and pulled up while the dish and membrane were placed against the maternal skin. Pulling up on the skin in this manner helped create a watertight seal as well as keeping the skin accessible so it could later be held in place while retrieving and replacing the uterus. Using fine forceps, the slit edge and its apposing skin edge were lifted to reveal the underlying uterine segment. Using a second pair of forceps, the uterine segment was gently exteriorized by grasping between implantations sites. At this stage, the transparent membrane was checked to ensure it was still adherent to the maternal skin suggesting an intact watertight seal.

The Petri dish was then supported by Plasticene blocks and filled with calcium and magnesium-free phosphate-buffered saline (PBS) (figure 8C). The PBS served as an ultrasound-coupling medium, maintained hydration of the uterus, and was used at room temperature to reduce normal resting myometrial activity (and therefore the associated gross movements of the implantation sites). The exposed uterine segment was short, ~1–3 implantation sites, which helped stabilize the segment during uterine activity and during microinjections. It was supported laterally by a semi-circular sheet of Silicone rubber (Silastic L RTV silicone rubber; Dow Corning [32]), which was submerged next to it inside the Petri dish on the side opposite that of the microinjector (figure 8C). This 'uterine stabilizer' was cast in a modified Petri dish and then bisected prior to use.

After microinjecting all sites as described in detail below, the maternal abdomen was closed using 6-0 silk, (Sofsilk™, United States Surgical Corporation [33]) using a continuous suture for the peritoneum and abdominal muscles and interrupted mattress sutures (3–4) for the skin. Anaesthesia was discontinued and the mouse was placed in a heated recovery chamber. Usually the mouse was awake and active within 5–10 minutes. No pregnant mice died or aborted following microinjection at E6.5 or E7.5 in over 100 procedures performed to date.

### High frequency ultrasound

We used an ultrasound biomicroscope (UBM) with a 40 MHz probe (Vevo-660™; VisualSonics) for real-time (34 frames/s) microvisualization of the conceptus and microinjection pipette. The 40 MHz probe has a spatial resolution of ~50 μm at 40 MHz [20]. The mouse was mounted on the stage of a rail system (VisualSonics), which permitted the operator to readily adjust the position of the mouse while maintaining the microinjection pipette within the transducer-imaging plane (figure 8A).

### Micropipette preparation

Microinjection pipettes were prepared using methods adapted from prior work [27]. Glass microcapillary tubes (3 1/2 "replacement tubes" Drummond Scientific Company, cat. # 3-000-203-G/X [34]) were pulled using a vertical puller (Narishige PB-7 [35]) to produce a long taper. Pipettes were broken using forceps or a scalpel blade in the narrowed region under a dissection microscope and the tip diameters were measured using the calibrated length scale in the eyepiece of the microforge (Narishige MF-9). Pipette tips with an outer diameter of approximately 50–80 μm and inner diameter of approximately 40–50 μm were gradually beveled to 20 degrees (figure 8B) using a continuously moistened grinder for 20 minutes (Narishige EG-4). Too little moisture resulted in a high incidence of tips blocked by particles following the grinding process. All tips were carefully inspected after sharpening to ensure the tip was clear and sharp (figure 8B).

The microinjection pipette was filled with mineral oil before connecting it to the microinjector using the manufacturer's instructions (Nanoject *II*; Drummond Scientific Co.). Once attached, excess oil was expelled. Green fluorescent beads (polystyrene latex, 3.0 ± 0.1 μm diameter, 2.5% solids in water; 1.68 × 10^3 ^particles/nl; Fluoresbrite™ YG Microspheres; Polysciences [36]) were diluted 2:1 in saline. With the aid of a stereomicroscope (Leica, MS5 [37]) and micromanipulator, the tip of the microinjection pipette was positioned in a 15 μL drop of the bead mixture and the mixture aspirated using the microinjector controls immediately prior to use. The filling procedure was visually monitored using the stereomicroscope to ensure no air was aspirated and that the pipette tip did not touch the bottom of the dish to prevent the tip from being blunted or broken. When the microinjection pipette was filled, the microinjector was transferred to the micromanipulator on the rail system, for alignment within the ultrasound scan plane.

### Microinjection procedure

The microinjection pipette was imaged in PBS prior to the experiment. It was aligned in the scan plane using the XYZ controls so that the tip was centred within the focal zone of the transducer (area of image with the greatest resolution). This position, 'the guide point', was recorded by the UBM and remained displayed on the monitor. The pipette was then retracted leaving the tip in PBS to prevent dehydration-induced obstruction of the lumen.

**Figure 9:**
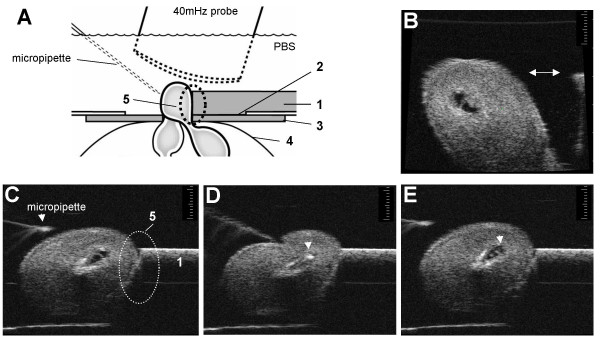
**Practical points to improve accuracy**. (A) Diagram of the optimum experimental set-up enlarged from that shown in figure 8. The uterine stabilizer (1) is cast in one of the modified Petri dishes so that it extends into the central hole (2). As a result the uterine stabilizer fits more closely to the transparent membrane (3) attached to the under surface of the petri dish and also creates a more secure seal with the skin of the mouse abdomen (4). This is important because it reduces the possibility of the exposed segment of uterine horn slipping between the under surface of the stabilizer and the transparent rubber membrane or the maternal skin during microinjections. Keeping the exposed uterus closely approximated to the uterine stabilizer (dotted oval labelled 5 in A &C) is very important in improving accuracy of microinjections. This improves stability of the uterine segment during microinjection allowing easier penetration of the micropipette through the thick uterine muscle enabling a more accurate placement of the tip of the microinjection pipette into the target region. (B) If the uterus is separated from the edge of the uterine stabilizer (double-headed arrow), the uterus will move away from the advancing needle and the target area of the conceptus will move out of the focal zone and/or field of view of the transducer reducing the accuracy of the microinjections. (C) The optimal position of the uterus relative to the uterine stabilizer is demonstrated. The alignment of the conceptus within the scan plane is also optimal. In this position three potential targets are easily accessible from a lateral approach thereby avoiding injury to the embryo caused by the microinjection pipette or the inadvertent deposition of fluorescent beads in other targets during needle insertion or withdrawal. (D) Placement of the microinjection pipette within the amniotic cavity (arrow). (E) Immediately after removal of the microinjection pipette, a small amount of echogenic material (fluorescent beads) can be seen within the amniotic cavity (arrow) which was not present prior to microinjection (compare with amniotic cavity in image B).

During experiments, implantation sites were moved into the scan plane using the XYZ controls on the mouse stage. The target region of the conceptus was positioned at the guide point displayed on the UBM's monitor, and then the microinjection pipette was advanced until the tip was visible within the target region. The injectate was then administered at a rate of 23 nL/s (Nanoject *II*; Drummond Scientific Company). Injected beads were echogenic when injected into cavities thereby permitting a visual impression of targeting success (figure 9E). Accuracy was improved by ensuring the uterine segment was directly adjacent to the 'uterine stabilizer' at the time of microinjection (figure 9A, C) and by the sharpness of the microinjection pipette, both of which minimized the movement of the uterus as the microinjection pipette advanced. Using a lateral approach to microinject the three-targeted regions also helped to improve accuracy. This approach avoids passage of the needle through non-targeted regions of the conceptus and the associated risk of mechanical injury or deposition of beads in these regions. The time of microinjection was coordinated with the spontaneous motion of the uterine segment (caused by uterine muscle activity) to optimise alignment of the uterine segment against the 'uterine stabilizer' and the path of the microinjection pipette to the target (figure 9B–D).

After injecting the 1–3 exposed sites, they were replaced and the uterine segment containing the next 1–3 implantation sites was carefully removed from the abdominal cavity, pulled gently through the water seal membrane into the PBS-filled Petri dish and was laid next to the uterine stabilizer. The patency of the microinjection pipette was confirmed before continuing.

We microinjected the targeted region within each site in both uterine horns (usually 10 to 16 sites) within 20 to 30 minutes (demonstration in additional files 3, 4, 5) using two operators. One operator loaded and discharged the needle and took notes, while the other performed surgery, operated the UBM, and positioned the microinjection pipette in the target area. Abnormal sites that appeared to contain embryos undergoing re-absorption had their positions noted but were not injected.

At necropsy, the localization of beads was visually evaluated using a stereomicroscope (Leica, MS5 [37]) with an attached universal light source (MAA-002 from BLS Ltd. [38]) in implantation sites that were dissected as previously described [21].

### Histology

Histology was performed on implantation sites that were immersion-fixed in 4% paraformaldehyde, dehydrated in alcohol, and paraffin embedded. Tissue was sectioned (5 μm) and stained with hematoxylin and eosin (H&E) to show general morphology of the implantation site for comparison with ultrasound images (figure 1B, D). In order to detect the fluorescent beads, 50 μm frozen sections of paraformaldehyde-fixed sites were imaged using an inverted microscope with a FITC filter to detect the fluorescent beads. Images showing the beads were superimposed on autofluorescent images of the tissue obtained using a CY-3 filter (tissue autofluoresces red/orange, figure 4) or using immunofluorescence to detect collagen 4 (basement membranes of blood vessels appear pink, figure 5B–D) and/or following counterstaining with 4', 6-Diamidino-2-phenylindole (DAPI; DNA fluoresces blue, figure 6B–D).

### Statistics

95% confidence intervals were used to determine statistical significance when comparing target accuracy between the different target sites at E6.5 and E7.5 and when comparing survival between the experimental and sham groups. Linear regression was used to calculate the average number of embryos at a given gestation in non-operated control pregnancies. P < 0.05 was considered significant.

## Authors' contributions

JS helped to design the experimental apparatus, performed the experiments, analyzed results, and drafted the manuscript. LB perfected and manufactured the microinjection pipettes and assisted in all experiments and dissections. MG selected the microinjection apparatus; helped perfect the microinjection pipettes, assisted in initial experiments, and assisted in manuscript preparation. JM assisted in initial experiments. DQ adapted the surgical procedures and assisted in initial experiments. NS assisted with statistical analysis of data. JK supervised JS in the Maternal Fetal Medicine training program and assisted in manuscript preparation. JR and SLA conceived the study, and participated in design and coordination. SLA assisted in manuscript preparation and revision.

## Supplementary Material

Additional File 1 Ultrasound of an E6.5 conceptus. This video clip shows a conceptus at E6.5 in a longitudinal view within the exteriorized uterus. The echogenic 'V' shaped ectoplacental cone region is on the left-hand side of the conceptus within the uterus. The common proamniotic and exocoelomic cavity is difficult to see because its diameter is near the resolution limit of the 40 MHz ultrasound transducer. The smallest divisions in the scale bar are 100 μm. The frame rate is 34 frames/second. Click here for file

Additional File 2 Ultrasound of an E7.5 conceptus. In this video clip the uterus is again exteriorized and a conceptus at E7.5 is seen in a longitudinal view. There are two distinct cavities visible. The cavity on the left is the amniotic cavity, which is separated from the adjoining exocoelomic cavity by the amniotic membrane. The allantois is easily seen traversing the exocoelomic cavity. Immediately to the right of the exocoelomic cavity is the echogenic ectoplacental region with the ectoplacental cleft/cavity just starting to become visible. The smallest divisions in the scale bar are 100 μm. The frame rate is 34 frames/second. Click here for file

Additional File 3 Demonstration of a microinjection of 13.8 nL of fluorescent beads into the amniotic cavity at E7.5. This video clip shows advancement of the microinjection pipette into the amniotic cavity at E7.5. The beads being echogenic are visible on ultrasound and can be seen exiting the tip of the pipette. The beads are visible within the cavity following removal of the microinjection pipette. The smallest divisions in the scale bar are 100 μm. The frame rate is 34 frames/second. Click here for file

Additional File 4 Demonstration of a microinjection of 13.8 nL of fluorescent beads into the exocoelomic cavity at E7.5. This video clip shows advancement of the microinjection pipette into the exocoelomic cavity at E7.5. The uterus is supported by the uterine stabilizer on the right of the screen and prevents the uterus moving away from the advancing microinjection pipette. The smallest divisions in the scale bar are 100 μm. The frame rate is 34 frames/second. Click here for file

Additional File 5 Demonstration of a microinjection of 13.8 nL of fluorescent beads into the ectoplacental cone region at E7.5. This video clip shows advancement of the microinjection pipette into the ectoplacental cone region at E7.5. The echogenicity of the tissue around and within the ectoplacental cone region makes it difficult to appreciate the injection of the beads as seen in the other video clips. The smallest divisions in the scale bar are 100 μm. The frame rate is 34 frames/second. Click here for file
